# The responsiveness of goal attainment scaling using just one goal in controlled clinical trials: an exploratory analysis

**DOI:** 10.1186/s41687-020-00196-8

**Published:** 2020-05-12

**Authors:** Lisa McGarrigle, Kenneth Rockwood

**Affiliations:** 1 Division of Geriatric Medicine, Department of Medicine, Dalhousie University and Nova Scotia Health Authority, 5955 Veterans’ Memorial Lane, Halifax, NS B3H 2E1 Canada; 2 DGI Clinical Inc., 1730 Market St, Halifax, NS B3J 3N9 Canada; 3 School of Health Sciences, Faculty of Biology, Medicine and Health, The University of Manchester, Manchester, M13 9PL UK; 4 Centre for Health Care of the Elderly, Nova Scotia Health Authority, 1421-5955 Veterans’ Memorial Lane, Halifax, Nova Scotia B3H 2E9 Canada

**Keywords:** Goal attainment, Responsiveness, RCT, Frailty, Dementia, Assessment

## Abstract

**Background:**

Goal Attainment Scaling (GAS) is an individualized outcome measure that allows the setting of personalized treatment goals. We compared the responsiveness of GAS when individuals set only one goal instead of the recommended three or more goals.

**Methods:**

We conducted exploratory analyses on data from two randomized controlled trials: the Video-Imaging Synthesis of Treating Alzheimer’s Disease (VISTA) (*n* = 130); and the Mobile Geriatric Assessment Team (MGAT) (*n* = 265). Independent t-tests and standardized response means (SRMs) were used to assess responsiveness of one- vs. multiple-goal GAS.

**Results:**

In VISTA, clinician-rated multiple-goal GAS detected higher goal attainment in the intervention group (*p* = 0.01; SRM = 0.48). One-goal GAS, whether rated by patients or by clinicians, did not detect differences in goal attainment between groups (patient: *p* = 0.56, SRM = 0.10; clinician: *p* = 0.10, SRM = 0.29). In MGAT, multiple-goal GAS (outcome goals: *p* < .001, SRM = 1.29; total goals: *p* < .001, SRM = 1.52) and one-goal GAS (outcome goals: *p* < .001, SRM = 0.89; total goals: *p* < .001, SRM = 0.75), detected significantly higher goal attainment in the intervention group.

**Conclusion:**

One-goal GAS detected significant change in response to a patient-centred, multi-domain care initiative. As such, in similar contexts, one-goal GAS may be an effective means of optimizing personalization and improving GAS feasibility through reduced administration time. However, it is not yet clear if one-goal GAS is responsive in the context of a pharmacological intervention and further research is recommended.

## Introduction

As people age, they accumulate health deficits, so that single-system illness is not commonly the norm. For complex patients who require complex interventions, goal-oriented patient care can offer an effective means of evaluating treatment outcomes [1]. A useful approach to the assessment of treatment efficacy in complex conditions involves determining how well a patient meets their personal goals across one or multiple domains (e.g., cognitive, functional, social, etc.). A patient-centred, goal-oriented approach to outcome measurement has several advantages, and thereby can complement traditional disease-specific measures, including simplified and effective shared decision-making, emphasis on individually, rather than universally, desired health states, and increased control for patients regarding their treatment options [2]. Goal Attainment Scaling (GAS) [3], is one such measure that allows patients and their clinicians to evaluate achievement of personalized treatment goals over time. The GAS process usually begins with a clinician-facilitated interview to identify areas of challenge that are of particular relevance to the patient and/or caregiver, and to set three or more goals, often weighted according to personal importance. Baseline status for each goal area is then established and outcomes considered better and worse than baseline are described to complete the 5-point GAS scale, ranging from + 2 (much better than expected) to − 2 (much worse than expected). The scale is then used at designated time intervals to assess the degree of goal attainment.

Although GAS was first developed over 50 years ago, it has not been used extensively in routine care, as its administration has been seen as cumbersome [4]. For this reason, efforts have been made to make GAS more feasible through the development of standardized symptom menus [1, 5] and goal inventories [6] to facilitate goal setting. A further challenge to the feasibility of GAS relates to the setting of multiple goals. GAS guidelines recommend setting three or more goals [7], however this may lead to longer administration times and, in some cases, reduced personalization. An example of this was encountered in a recent hemophilia study [5], where many participants, especially adolescent males, objected to having to set multiple goals, instead preferring to focus on only one target goal that was especially important to them. Additionally, a recent study investigating the feasibility of GAS for use in clinical care [8] utilized a 41-item goal inventory to assist in goal setting, while also limiting the number of goals that could be set by each patient-caregiver dyad to one, based on their highest ranking goal. Although the reason for focusing on only one goal was not made explicit, it likely simplified GAS administration and the approach was deemed feasible in the context of clinical care of persons with dementia. Even so, the considerable simplification of the GAS formula when only one goal is set may impact on psychometric indicators where variability is key, such as responsiveness, often referred to as sensitivity to change [9]. If the use of one-goal GAS limits the responsiveness of the evaluation, this is something that patients and clinicians should be made aware of. If responsiveness is impacted by selecting only one goal, this could also have implications for clinical trials using the measure to detect change. A useful test of this would be to compare GAS responsiveness, calculated based on one vs. multiple goals, in the context of an RCT. Here we report on the gains and losses in responsiveness in relation to the amount of GAS goals set (one or multiple) in two RCTs: one employed a pharmacological intervention in individuals with Alzheimer’s disease, and the other evaluated a patient-centred care initiative in older adults living with frailty. We chose to investigate responsiveness in two different trials as aspects of the GAS methodology often vary across studies (e.g., patient, caregiver, or clinician raters; goal weighting). As such, it was of interest to investigate GAS responsiveness in the context of variable methodologies in order to enhance the generalizability of our findings.

## Methods

### The study samples

Exploratory analyses were conducted on data from two randomized controlled trials in which GAS was the primary outcome measure: the Video-Imaging Synthesis of Treating Alzheimer’s Disease (VISTA) study, a 4-month, double-blind, placebo-controlled trial of galantamine in community-dwelling, mild to moderate AD patients; and the Mobile Geriatric Assessment Team (MGAT) study, a randomized, controlled trial that examined the effect that recommendations based on the Comprehensive Geriatric Assessment (CGA) had on achieving patient-centred goals in rural, community-dwelling frail older adults.

**Fig. 1:**
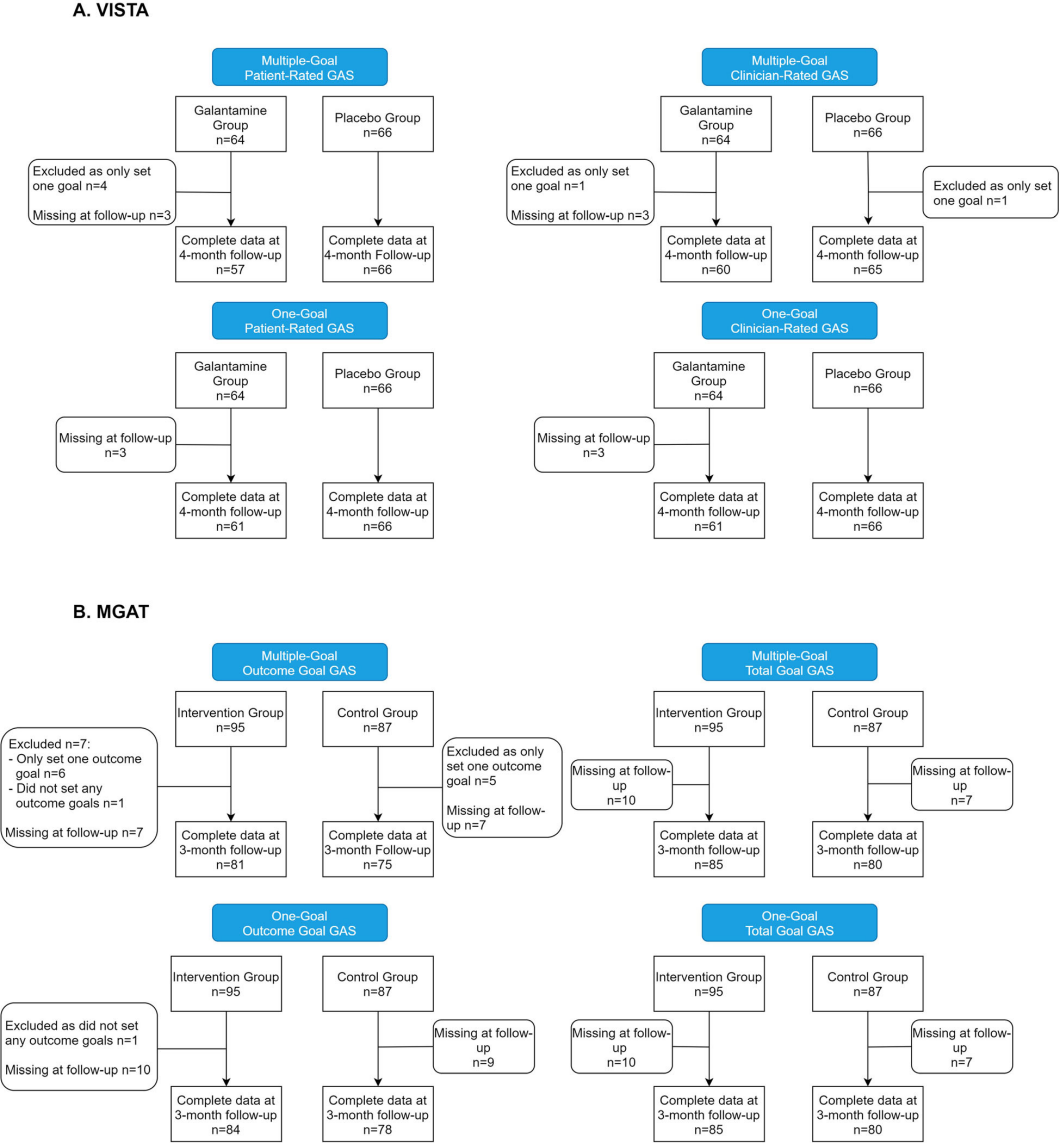
Flow chart of VISTA and MGAT subjects included in multiple- and one-goal GAS analyses. In VISTA, analyses were conducted on patient-rated GAS scores and clinician-rated GAS scores. In MGAT, analyses were conducted on outcome goal scores (goals directed towards outcomes of care) and total goal scores (goals directed towards outcomes, but also those focused on prevention or the completion of a particular process, which may be easier to achieve)

As described elsewhere [10], the VISTA sample consisted of 159 patients with mild to moderate Alzheimer’s disease; 23 did not meet inclusion criteria, five withdrew before assessment, and one died before assessment. Of the remaining 130 participants, 64 were randomized to the galantamine group and 66 to the placebo group. The MGAT sample, also described elsewhere [11] consisted of 265 older patients referred to Geriatric Medicine; 54 refused or withdrew before assessment, 27 did not meet inclusion criteria, and two died before assessment. Of the remaining 182 participants, 95 were randomized to intervention and 87 to the control group. A comparison of the key characteristics of the VISTA and MGAT studies is provided in Table 1. VISTA and MGAT subjects with complete data at follow-up for both multiple- and one-goal GAS analyses are illustrated in Fig. 1. Note that the smaller sample sizes in this report compared with the original reports [9, 10] reflect the exclusion of patients who had only set one goal from the multiple-goal analyses (six in VISTA and eleven in MGAT Outcome-Goal GAS). Likewise, the responsiveness statistics, though similar to the original reports as here (below), are not exactly the same.

**Table 1:** Characteristics of the VISTA and MGAT studies

	**Video-Imaging Synthesis of Treating Alzheimer’s Disease (VISTA)**	**Mobile Geriatric Assessment Team (MGAT)**
**Design**	Double-blind, placebo controlled RCT	Single-blind RCT – Assessments were performed by healthcare professionals blind to patient status
**Participants**	Community-dwelling adults with mild to moderate Alzheimer’s Disease (*n* = 130)	Rural, community-dwelling, frail elderly patients (*n* = 182)
**Intervention**	Patients were randomized to receive either galantamine (16–24 mg/d) or a placebo for a 4-month period	Patients were randomized to receive either usual care by family physicians or care in which physician efforts were supplemented by a specialized geriatric intervention over a 3-month period
**Primary Outcome Measure**	Goal Attainment Scaling (GAS)	Goal Attainment Scaling (GAS)
**GAS Methodology**	Assessment: Two independent GAS assessments were conducted at each time-point. - Patient/caregiver-rated GAS was assessed in a clinician-facilitated interview - Clinician-rated GAS was assessed following interviews with patients and/or caregivers.	Assessment: Patient-rated GAS was assessed in a clinician-facilitated interview both at baseline and three-month follow-up
	Goal setting: Goals were freely selected based on symptoms considered important to the patient	Goal setting: Participants selected goals based on a list of 42 symptoms or problems identified by the Comprehensive Geriatric Assessment (CGA)
	Goal weighting: Patients and caregivers ranked (weighted) their goals in order of importance. Clinicians did not rank goals, so all were assigned a weighting of ‘1’	Goal weighting: As goals were not ranked, all were assigned a weighting of ‘1’
	Baseline scores: Baseline raw GAS scores set at ‘0’	Baseline scores: Baseline raw GAS scores were set at ‘-1’, except for a small number of participants (*n* = 5). These participants were assigned baseline scores of ‘-2’ based on the clinical judgement that their situation could not plausibly deteriorate.
	Goal selection for one-goal GAS: One-goal GAS scores were derived for patients/caregivers using their highest-ranking goal One-goal GAS scores were derived for clinicians by selecting the first goal listed for each patient	Goal selection for one-goal GAS: One-goal GAS scores were derived by randomly selecting a goal for each participant using a custom excel macro
	Number of Goals Set: The median number of goals set was 3 (range 1–6)	Number of Goals Set: The median number of goals set was 5 (range 2–15)

### Calculating multiple-goal and one-goal GAS

GAS [3] was the primary outcome measure in both trials. In VISTA, two independent GAS assessments were conducted at baseline and four-month follow-up: one by patients/caregivers in a clinician-facilitated interview (patient-rated GAS); and the other by clinicians following interviews with patients/caregivers (clinician-rated GAS). At baseline, between one and six goals were set for both patient- and clinician-rated GAS, and patients/caregivers ranked (weighted) their goals by importance. GAS baseline status was anchored at ‘0’. Prior to calculating multiple-goal GAS, patients who only set one goal (*n* = 4 for patient-rated GAS; *n* = 2 for clinician-rated GAS) were excluded (Fig. 1). Patient- and clinician-rated multiple-goal GAS scores were calculated using the following formula:

$$ T=50+\frac{10\sum {w}_i{x}_i}{\sqrt{\left(1-\rho \right)\sum {w}_i^2+\rho {\left(\sum {w}_i\right)}^2}} $$

where T is the composite score, *w*_i_ is the weight assigned to the *i*th goal, *x*_i_ is the numerical value (− 2 to + 2) of the attainment level of the *i*th goal, and *ρ* is the estimated correlation between goal scores, assumed to be constant at .3 [3].

One-goal GAS scores were derived for patients/caregivers using their highest-ranking goal. As goals were not ranked for clinician-rated GAS (all were given a weighting of 1), one-goal GAS scores were calculated by selecting the first goal listed for each patient. Patient- and clinician-rated one-goal GAS scores were calculated using the following, simplified GAS formula:

$$ GAS=50+(10x) $$

where *x* is the numerical value (− 2 to + 2) of the attainment level. A detailed description of how the one-goal GAS formula was derived is provided elsewhere [9].

In MGAT, patient-rated GAS was assessed in a clinician-facilitated interview both at baseline and three-month follow-up. All participants set between two and 15 goals, selected from a list of 42 symptoms or problems identified by the CGA. In most cases, GAS baseline status was anchored at ‘-1’. However, a small number of MGAT participants (*n* = 5) were assigned baseline scores of ‘-2’, based on the clinical judgement that their situation could not plausibly deteriorate. As in the VISTA study, most goals were directed toward outcomes of care (*outcome* goals), however there were occasions where goals focused on prevention or the completion of a particular process. As the latter could be considered easier to achieve, we chose to conduct our initial analysis on outcome goals only, before then including *prevention* and *process* goals for a total goal analysis. Prior to calculating outcome-goal GAS for multiple-goals, patients who only set only one outcome goal (*n* = 11) were excluded (Fig. 1). As goals were not ranked (all were assigned a weighting of 1), one-goal GAS was calculated for participants by randomly selecting a goal for each participant using a custom excel macro. Multiple- and one-goal GAS for both outcome and *total* goals were calculated using the GAS formulae presented above.

### Analysis

As GAS is a change score, standardized response means (SRMs) [12] were used to assess clinical detectability and responsiveness to the intervention at 4 months in VISTA and 3 months in MGAT. SRMs were calculated by dividing the absolute difference between the groups (intervention mean change in GAS scores minus control mean change) by the pooled standard deviation of the change. Cohen’s effect size criteria [13] were used to interpret results, with a value of 0.2 considered “small”, 0.5 “medium”, and 0.8 “large”. Independent t-tests were conducted to evaluate intervention efficacy by comparing the intervention and control groups on their mean change in GAS scores at follow-up. To ensure the robustness of the one-goal GAS findings based on randomly selected goals, a sensitivity analysis was conducted by repeating SRM calculations and t-tests with a separate random selection of goals from both VISTA and MGAT.

## Results

### Sample characteristics

In VISTA, participants were aged between 50 and 93 years, 48 (37%) were male and 82 (63%) were female, and all were diagnosed with mild to moderate Alzheimer’s disease. In MGAT, participants were aged between 66 and 97 years, 78 (43%) were male and 104 (57%) were female, and all were frail older adults referred to geriatric medicine. Sample characteristics for intervention and control groups are provided in Table 2.

**Table 2:** Sample characteristics at baseline

	**VISTA**		**MGAT**	
	Intervention *n = 64*	Control *n = 66*	Intervention *n = 95*	Control *n = 87*
Age in years, mean (SD, range)	76 (8, 50–93)	78 (8, 55–91)	81 (7, 66–97)	82 (7, 66–97)
Male sex, no. (%) of patients	23 (36)	25 (38)	41 (43)	37 (43)
MMSE score, mean (SD, range)	20.8 (3.3, 11–26)	19.9 (4.2, 9–27)	22.7 (6.3, 6–30)	22.9 (7.1, 0–30)

### Responsiveness

**Fig. 2:**
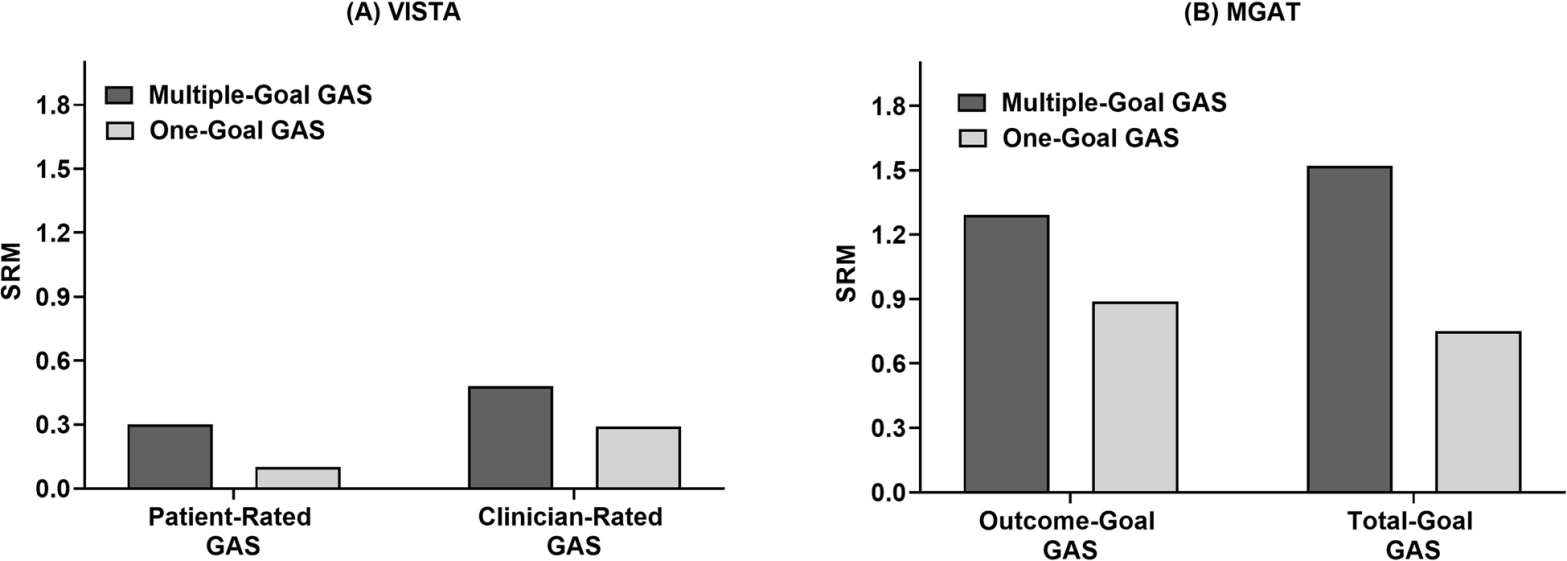
Responsiveness gains and losses in relation to number of goals set in VISTA and MGAT

SRM gains and losses in relation to the number of goals set (one or multiple) in VISTA and MGAT are illustrated in Fig. 2. In VISTA, clinician-rated multiple-goal GAS detected higher goal attainment in the intervention group (absolute difference between groups = 4.5, *p* = 0.01; SRM = 0.48), whereas patient-rated multiple-goal GAS did not (absolute difference = 2.5, *p* = 0.15; SRM = 0.26). One-goal GAS, whether rated by patients or clinicians, did not detect significant differences in goal attainment between groups, although responsiveness was within the rubric of a small effect size (patient: absolute difference = 1.0, *p* = 0.56, SRM = 0.10; clinician: absolute difference = 2.6, *p* = 0.10, SRM = 0.29). GAS scores and treatment response in VISTA are presented in Table 3.

**Table 3:** VISTA patient- and clinician-rated GAS scores and treatment response for multiple- and one-goal GAS

	**Control**			**Intervention**			*p*	*SRM*	*SD*_*p*_
	*n*	*Pre Mean (SD)*	*Post Mean (SD)*	*n*	*Pre Mean (SD)*	*Post Mean (SD)*			
**Multiple-Goal GAS**									
Patient-rated GAS	66	50.0 (0.0)	52.2 (9.0)	57	50.0 (0.0)	54.7 (10.2)	.15	0.26	9.6
Clinician-rated GAS	65	50.0 (0.0)	50.6 (9.2)	60	50.0 (0.0)	55.1 (9.5)	.01	0.48	9.3
**One-Goal GAS**									
Patient-rated GAS	66	50.0 (0.0)	52.1 (9.7)	61	50.0 (0.0)	53.1 (9.4)	.56	0.10	9.6
Clinician-rated GAS	66	50.0 (0.0)	51.3 (8.9)	61	50.0 (0.0)	53.9 (8.5)	.10	0.29	8.7

Note. SRMs were derived as the mean difference between treatment groups divided by the pooled standard deviation of their change. *SD*_*p*_ Pooled Standard Deviation of Change

In MGAT, *outcome* multiple-goal GAS detected significantly higher goal attainment in the intervention group (mean difference = 8.0, *p* < .001; SRM = 1.29). *Outcome* one-goal GAS also detected significantly higher goal attainment in the intervention group, although to a lesser magnitude than multiple-goal GAS (mean difference = 5.7, *p* < .001; SRM = 0.89). *Total* multiple-goal GAS detected significantly higher goal attainment in the intervention group (mean difference = 7.9, *p* < .001; SRM = 1.52), as did *total* one-goal GAS, but again, to a lesser magnitude than multiple-goal GAS (mean difference = 4.2, *p* < .001; SRM = 0.75). GAS scores and treatment response in MGAT are presented in Table 4.

**Table 4:** MGAT patient- and clinician-rated GAS scores and treatment response for multiple- and one-goal GAS

	**Control**			**Intervention**			*p*	*SRM*	*SD*_*p*_
	*n*	*Pre Mean (SD)*	*Post Mean (SD)*	*n*	*Pre Mean (SD)*	*Post Mean (SD)*			
**Multiple Goal GAS**									
Outcome Goal GAS	75	36.1 (1.4)	40.6 (5.6)	81	35.8 (1.3)	48.1 (6.7)	<.001	1.29	6.2
Total Goal GAS	80	34.9 (0.8)	38.7 (4.1)	85	34.8 (1.0)	46.4 (5.9)	<.001	1.52	5.2
**One Goal GAS**									
Outcome Goal GAS	78	39.8 (1.5)	43.1 (5.9)	84	39.8 (1.5)	48.8 (7.1)	<.001	0.89	6.3
Total Goal GAS	80	39.9 (1.1)	42.8 (5.6)	85	40.0 (0.0)	47.1 (5.7)	<.001	0.75	5.6

### Sensitivity analysis for one-goal GAS

To ensure the robustness of the one-goal GAS effect sizes that were calculated based on randomly selected goals (clinician-rated GAS in VISTA; both *outcome* and *total* goal GAS in MGAT), SRM calculations and t-tests were repeated with an additional random selection of goals from each study sample.

Repeated analysis in VISTA suggests that the SRM for clinician-rated GAS is small and there was no significant difference in one-goal attainment between the intervention and control group at 4 months. Repeated analyses in MGAT found SRMs indicating moderate to large effect sizes, and significantly higher goal attainment was detected in the intervention groups for both *outcome* and *total* goal GAS at 3 months. These findings support the robustness of the original one-goal GAS findings (Table 5).

**Table 5:** One-Goal GAS sensitivity analysis^a^

	**Control**			**Intervention**			*p*	*SRM*	*SD*_*p*_
	*n*	*Pre Mean (SD)*	*Post Mean (SD)*	*n*	*Pre Mean (SD)*	*Post Mean (SD)*			
**VISTA**									
Clinician-Rated GAS	66	50.0 (0.0)	50.5 (8.7)	61	50.0 (0.0)	52.6 (9.1)	.19	0.24	8.9
**MGAT**									
Outcome Goal GAS	76	40.0 (0.0)	43.8 (6.5)	84	39.9 (1.0)	48.8 (6.0)	<.001	0.82	6.3
Total Goal GAS	79	40.0 (0.0)	42.5 (4.8)	84	40.0 (0.0)	47.6 (7.3)	<.001	0.82	6.2

^a^One-Goal GAS analyses (clinician-rated GAS in VISTA and both *outcome* and *total* goal GAS in MGAT) repeated with a new set of randomly selected goals

In addition, as the VISTA trial involved a pharmacological intervention, it is possible that some of the randomly selected goals for both patient- and clinician-rated GAS were not as likely to be achieved as others. For example, the goals in VISTA can be broadly grouped into five domains: behavior, cognition, function, social, and leisure. Goals relating to cognition and function made up the majority of goals set (see Appendix 1 for the proportion of goals set per domain; see Appendix 2 for examples of the types of goals within each domain). Certain goals related to function, such as bathing, dressing or meal preparation, may require caregiver assistance and as such, may not be as responsive to the pharmacological intervention as those related to cognition (e.g., attention; disorientation) and behaviour (e.g., agitation; delusions). To further interrogate this, SRM calculations and t-tests were repeated after removing all goals relating to function.

Repeated analysis in VISTA suggests that the SRM’s for both patient- and clinician-rated GAS are small (0.14 and 0.21, respectively) and there was no significant difference in one-goal attainment between the intervention and control group at 4 months. These findings also support the robustness of the original one-goal GAS findings.

## Discussion

The responsiveness gains and losses incurred in relation to the amount of GAS goals set (one or multiple) was formally tested using RCT data from a pharmacological intervention in individuals with Alzheimer’s disease (VISTA), and a patient-centred care initiative in frail older adults (MGAT). In both trials, gains in effect sizes were apparent for multiple-goal GAS compared to one-goal GAS, although in MGAT all were considered as large according to Cohen’s criteria. Further, one-goal GAS detected a significant treatment effect in MGAT, but not VISTA. In contrast, multiple-goal GAS detected a treatment effect in both trials, although in VISTA a significant effect was only observed for clinician-rated GAS.

Sensitivity analyses confirmed that differences in responsiveness of one-goal GAS in MGAT and VISTA were unlikely to be due to the random selection of one goal for analysis. Sensitivity analyses also suggested that the type of goals selected for one-goal GAS analysis in VISTA did not impact on responsiveness. The large SRMs and significant treatment effect observed for one-goal GAS in MGAT may be due in part to the larger sample, and as such, the small effect size for clinician-rated one-goal GAS in VISTA merits further investigation using data from a larger clinical trial. The significant MGAT findings may also be due to the specialized geriatric intervention, designed to target multiple medical and social problems in frail older adults. In addition to differing interventions, the GAS methodology varied between studies in a number of ways, including the method of goal selection, as highlighted in Table 1. These differences may have impacted GAS responsiveness. For instance, in MGAT, participants selected goals based on a list of 42 symptoms or problems identified by the CGA. As such, the CGA-based intervention was primed to target these kinds of problems which could explain why large improvements were seen in the intervention group. Furthermore, assessors were blinded to the intervention, whereas patients were not, and this may have influenced responsiveness to the intervention, leading to larger effect sizes. It is also possible that differences in responsiveness are due to the study samples (frailty vs. mild to moderate Alzheimer’s disease). However, frail older adults share many attributes with mild to moderate dementia patients in that both groups have varied symptoms across multiple domains. The majority of VISTA participants set goals relating to cognitive and functional outcomes, and the top three outcome goals set in MGAT (mobility, memory, and mood) also relate to cognitive and functional outcomes. Furthermore, VISTA and MGAT participants were closely matched with regard to degree of cognitive impairment (mean VISTA MMSE score = 20.3; mean MGAT MMSE score = 22.8).

Multiple-goal GAS has demonstrated responsiveness across a range of settings, including geriatric rehabilitation in frailty [14–17], clinical trials in dementia [10, 18], neurorehabilitation in acquired brain injury [19], pediatric rehabilitation in cerebral palsy [20], and inpatient rehabilitation in multiple sclerosis [21]. The responsiveness of multiple-goal GAS is likely due to the range of domains assessed and its ability to detect small changes in areas important to the individual that may not be addressed by standard measures [4]. This makes GAS well suited to the assessment of clinically meaningful change in conditions where symptom heterogeneity is common [1]. Findings from this study support the responsiveness of one-goal GAS in the context of a patient-centred intervention designed to target a broad range of domains. As such, one-goal GAS may be an effective means of improving the feasibility of GAS by reducing the number of target goals, and consequently, GAS administration time. Additionally, one-goal GAS can potentially optimize personalization by allowing patients to focus on only one goal if they so choose. Even so, gains in responsiveness were noted when more than one goal was used to calculate GAS scores. It is not yet clear if one-goal GAS is responsive in the context of a pharmacological intervention and we recommend further research in a larger sample before implementing one-goal GAS in this context. Still, it is interesting to note that patients (and/or caregivers) preferring to focus on a single goal was evident in the VISTA study, being present in almost 5% of patients.

## Conclusion

In exploratory analyses in each of two settings - a patient-centred multidisciplinary intervention and a clinical trial of an anti-dementia drug, Goal Attainment Scaling based on only one goal was less responsive to the intervention than was traditional multiple-goal GAS. Still, one-goal GAS offers a promising means of improving the feasibility of GAS, but also optimizing personalization by allowing patients to focus on only one goal if they so choose. This seems more likely in the context of a multi-domain, patient-centred, targeted intervention, where a statistically significant difference was still demonstrated with one-goal GAS, than in the drug trial. Further research on the responsiveness of one-goal GAS, especially in the context of a pharmacological intervention, is necessary.

## Data Availability

The datasets used and/or analysed during the current study are available from the corresponding author on reasonable request.

## Appendix 1

### Number and percentage of goals set by domain in VISTA

**Table:**

Domain	One-Goal GAS			Multiple-Goal GAS		
	*Control (%)*	*Treatment (%)*	*Total (%)*	*Control (%)*	*Treatment (%)*	*Total (%)*
Patient-Rated GAS						
Behaviour	14 (21.2)	9 (14.1)	23 (17.7)	46 (20.0)	36 (16.7)	82 (18.4)
Cognition	35 (53.0)	34 (53.1)	69 (53.1)	101 (43.9)	96 (44.7)	197 (44.3)
Function	12 (18.2)	15 (23.4)	27 (20.8)	56 (24.3)	48 (22.3)	104 (23.4)
Leisure	3 (4.5)	5 (7.8)	8 (6.2)	14 (6.1)	18 (8.4)	32 (7.2)
Social	2 (3.0)	1 (1.6)	3 (2.3)	13 (5.7)	17 (7.9)	30 (6.7)
Clinician-Rated GAS						
Behaviour	26 (39.4)	17 (26.6)	43 (33.1)	44 (22.8)	38 (20.1)	82 (21.5)
Cognition	6 (9.1)	11 (17.2)	17 (13.1)	48 (24.9)	57 (30.2)	105 (27.5)
Function	33 (50.0)	33 (51.6)	66 (50.8)	64 (33.2)	57 (30.2)	121 (31.7)
Leisure	1 (1.5)	3 (4.7)	4 (3.1)	21 (10.9)	21 (11.1)	42 (11.0)
Social	–	–	–	16 (8.3)	16 (8.5)	32 (8.4)

## Appendix 2

### Types of Goals Selected in VISTA

**Table:**

Goal Domain (Number, % of total goals)	Goal Type	Number
Patient-Rated One-Goal GAS		
Behaviour (23, 17.7%)	Agitation/Irritation	5
	Appetite	1
	Delusions	4
	Hoarding/Hiding	1
	Low Mood	5
	Repetitive Behaviour	1
	Sleep Disturbances	6
Cognition (69, 53.1%)	Attention/Concentration	5
	Disorientation -Time	10
	Disorientation-Place/Space	3
	Expressive Language Difficulty	2
	Following Instructions/Multi-Tasking	2
	Judgement/Problem Solving	1
	Misplacing	6
	Physical Complaints	1
	Prospective Memory Impairment	3
	Recalling Recent Events	11
	Remembering Names	10
	Repetition	13
	Word Finding Difficulty	2
Function (27, 20.8%)	ADL-Bathing	1
	ADL-Dressing	4
	ADLs	2
	IADL-Dishes	1
	IADL-Driving	1
	IADL-Finances	1
	IADL-Household Chores/Repairs	1
	IADL-Meal Preparation	2
	IADL-Medication	4
	IADL-Operating Appliances/Devices	2
	IADL-Telephone Use	1
	Independence	2
	Physical Complaints	5
Leisure (8, 6.2%)	Loss of Hobbies/Pastimes	8
Social (3, 2.3%)	Social Interaction/Activities	3
Clinician-Rated One-Goal GAS		
Behaviour (43, 33.1%)	Agitation/Irritation	21
	Anxiety	8
	Apathy/Initiative	3
	Appetite	5
	Delusions	2
	Flat Affect	1
	Inappropriate Behaviour	1
	Low Mood	2
Cognition (17, 13.1%)	Comprehension	2
	Disorientation -Time	1
	Disorientation-Place/Space	3
	Expressive Language Difficulty	1
	Following Instructions/Multi-Tasking	4
	Misplacing	2
	Prospective Memory Impairment	2
	Recent Memory Impairment	1
	Repetition	1
Function (66, 50.8%)	ADL-Bathing	9
	ADL-Dressing	11
	ADL-Personal Hygiene	3
	ADLs	1
	Apraxia	1
	IADL-Dishes	1
	IADL-Driving	3
	IADL-Finances	2
	IADL-Household Chores/Repairs	7
	IADL-Meal Preparation	11
	IADL-Medication	7
	IADL-Telephone Use	3
	IADLs	2
	Independence	4
	Physical Complaints	1
Leisure (4, 3.1%)	Loss of Hobbies/Pastimes	4

## Abbreviations

CGA: Comprehensive Geriatric Assessment
GAS: Goal Attainment Scaling
MGAT: Mobile Geriatric Assessment Team
MMSE: Mini Mental State Examination
RCT: Randomized Controlled Trial
SRM: Standardized Response Mean
VISTA: Video-Imaging Synthesis of Treating Alzheimer’s Disease
